# Functional near‐infrared spectroscopy measures of neural activity in children with and without developmental language disorder during a working memory task

**DOI:** 10.1002/brb3.2895

**Published:** 2023-01-27

**Authors:** Allison S. Hancock, Christopher M. Warren, Tyson S. Barrett, David A. E. Bolton, Ronald B. Gillam

**Affiliations:** ^1^ Department of Psychology Utah State University Logan Utah USA; ^2^ Department of Kinesiology and Health Sciences Utah State University Logan Utah USA; ^3^ Department of Communicative Disorders and Deaf Education Utah State University Logan Utah USA

**Keywords:** auditory working memory, developmental language disorder, dorsolateral prefrontal cortex, fNIRS, inferior parietal lobule, *N*‐back

## Abstract

**Introduction:**

Children with developmental language disorder (DLD) exhibit cognitive deficits that interfere with their ability to learn language. Little is known about the functional neuroanatomical differences between children developing typically (TD) and children with DLD.

**Methods:**

Using functional near‐infrared spectroscopy, we recorded oxygenated hemoglobin (O_2_hb) concentration values associated with neural activity in children with and without DLD during an auditory *N*‐back task that included 0‐back, 1‐back, and 2‐back conditions. Analyses focused on the left dorsolateral prefrontal cortex (DLPFC) and left inferior parietal lobule (IPL). Multilevel models were constructed with accuracy, response time, and O_2_hb as outcome measures, with 0‐back outcomes as fixed effects to control for sustained attention.

**Results:**

Children with DLD were significantly less accurate than their TD peers at both the 1‐back and 2‐back tasks, and they demonstrated slower response times during 2‐back. In addition, children in the TD group demonstrated significantly greater sensitivity to increased task difficulty, showing increased O_2_hb to the IPL during 1‐back and to the DLPFC during the 2‐back, whereas the DLD group did not. A secondary analysis revealed that higher O_2_hb in the DLPFC predicted better task accuracy across groups.

**Conclusion:**

When task difficulty increased, children with DLD failed to recruit the DLPFC for monitoring information and the IPL for processing information. Reduced memory capacity and reduced engagement likely contribute to the language learning difficulties of children with DLD.

## Graphical abstract

Using fNIRS, we recorded oxygenated hemoglobin (O_2_hb) concentration values in children with and without DLD during an auditory *N*‐back task that included 0‐back, 1‐back, and 2‐back conditions. Typically developing children displayed sensitivity to working memory load in the frontal and parietal areas. Children with developmental language disorders demonstrated difficulties in engaging frontal parietal areas related to working memory. Higher O_2_hb concentration values in the left dorsolateral prefrontal cortex predicted better task accuracy across groups.

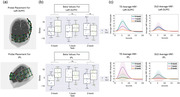

## 1 INTRODUCTION

Children with developmental language disorder (DLD) have impairments in language comprehension and production that are not attributable to conditions such as hearing loss or autism or to extenuating circumstances, such as lack of exposure to language (Bishop, 2017; Gillam et al., 2017). DLD affects one in 15 children (Norbury et al., 2016) and is a life‐long condition in which language deficits contribute to negative social, academic, and/or vocational consequences into adulthood (Arkkila et al., 2008; Clegg et al., 2005; Conti‐Ramsden et al., 2018). While the specific cause of DLD is unknown, the condition has been linked to a variety of phenomena, including genetic influences and deficits in cognitive functions (Conti‐Ramsden & Durkin, 2015). The influence of genetic factors is supported by high heritability rates in which families with a history of DLD have an estimated incidence of approximately 20%–40% compared to 4% in the general population (Choudhury & Benasich, 2003). DLD has been linked to specific single‐nucleotide polymorphisms such as FOXP2 (Fisher, 2005) and ATP2C2 (Martinelli et al., 2021). Commonly reported deficits in cognitive functions that are associated with DLD are reduced verbal working memory (WM) capacity (Montgomery et al., 2019) and procedural memory deficits (Ullman et al., 2020). In the present study, we examine WM performance in concert with cortical hemoglobin concentration measures in children with and without DLD to investigate how neural activity may differ between children with and without DLD.

The domain‐general account of language learning posits that cognitive mechanisms such as memory and attention play an integral role in learning and maintaining language (Chow et al., 2021; Montgomery et al., 2021). Consistent with this account, many behavioral studies have shown that children with DLD have limited WM capacity (Archibald & Gathercole, 2006; Gillam et al., 1995; Montgomery & Evans, 2009; Gray et al., 2019), reduced sustained attention (Finneran et al., 2009; Montgomery et al., 2009; Spaulding et al., 2008), and slower processing speeds than their typically developing (TD) peers (Leonard et al., 2007; Miller et al., 2001). These children may show deficiencies in one or many cognitive processes (e.g., WM, attention, processing speed, and any combination of the three) that can negatively affect their ability to acquire and use language (Bishop, 2006; Gillam et al., 2021). Children with DLD that demonstrate diminished WM capacity have difficulty with sentence comprehension, understanding grammatical rules, and learning new words (Montgomery, 2003; Montgomery et al., 2009, 2010). Controlled attention allows a person to filter out irrelevant information and focus on relevant information. Individuals with limited attentional control are often overloaded with irrelevant information (Awh & Vogel, 2008; Cowan et al., 2005; deBettencourt et al., 2019; Magimairaj & Montgomery, 2013). Slowed processing primarily interferes with language comprehension. In order to understand sentences, listeners need to switch between retrieving lexical knowledge and world knowledge while building a complete representation of what is being said. Incoming information that is not processed quickly enough is vulnerable to decay or interference, resulting in incomplete representations of what was said (Leonard et al., 2007). While there is wide agreement that cognitive skills are impaired in a DLD population, there is less consensus on some of the specific details of how it impacts language.

The limited capacity theory is a specific philosophical orientation within the general domain‐general model of language. According to this theory, children with DLD are affected by limitations in processing, storing, and retrieving information. These processing limitations negatively affect nonverbal intellectual abilities (Gallinat & Spaulding, 2014), leaving children especially vulnerable in language learning contexts that pose higher cognitive and linguistic demands (Weismer et al., 2005; Im‐Bolter et al., 2006). This finding is congruent with the work of Gillam et al. (2021), Leonard et al. (2013), Montgomery et al. (2018), and Robertson (2009), who have identified links between poor storage of previously processed linguistic material and sentence comprehension in a DLD population.

Cowan's embedded‐processes model of WM (Cowan, 2015, 1999, 2017) is consistent with the basic tenets of the limited capacity theory of DLD. According to the embedded‐processes model, WM is the ability to access and hold a limited amount of information in the focus of attention for use in cognition and language (Cowan et al., 2021). WM requires focused attention to activate a portion of long‐term memory. The focus of attention is limited in capacity and, as a result, affects the amount of possible information that is readily accessible to the learner at any given time (Cowan, 2014). Cognitive schemas found in long‐term memory contain organized information about experiences, thoughts, and behaviors and facilitate recoding of information into chunks that are easier to store and retrieve (Ericsson & Kintsch, 1995).

Children with DLD have highly variable WM profiles, meaning some children will evidence deficits in one or two aspects of WM (e.g., phonological loop, visual–spatial sketchpad, episodic buffer, central executive, memory updating, inhibition, etc.), while others may present deficiencies in multiple aspects of WM (Adams et al., 2018; Archibald, 2017; Gray et al., 2019). Although much has been learned about WM in children with DLD, identifying WM deficits and how they vary remains an important issue.

Measuring patterns of neural activity in children with DLD during a WM task can shed light on the cognitive abilities and associated neural processes that may differ in children with DLD and TD children. Lee and colleagues conducted two recent functional magnetic resonance imaging (fMRI) studies on a cohort of young adults with DLD and a cohort of adolescents with DLD using a cross‐sectional design. Both studies found that age‐related changes in white matter structures in the corticostriatal, dorsal, and ventral pathways, as quantified by fractional anisotropy, gradually emerged across time for participants with typical language abilities but not for participants in the DLD group. These biological changes were associated with multiple factors, including environmental, cognition, and language differences. Therefore, it was suggested that to understand the scope of DLD fully, researchers should employ multisystem methods that simultaneously investigate neural, cognitive, and linguistic abilities (Lee, Dick, et al., 2020; Lee, Nopoulos, et al., 2020).

### 1.1 *N*‐back tasks

*N*‐back is a useful paradigm for assessing WM because it measures the ability to store, manipulate, and update items in memory while inhibiting irrelevant information (Kane et al., 2007; Rottschy et al., 2012). In *N*‐back tasks, participants must recall a stimulus that was seen and/or heard “*N*” trials before a recall cue. A key finding of *N*‐back studies is the reliable decline in task performance as *N* increases (Braver et al., 1997; Ewing & Fairclough, 2010; Gajewski et al., 2018). In some *N*‐back experiments, researchers include a 0‐back condition, in which participants are asked to indicate when they hear a prespecified target within the presentation stream. Zero‐back requires sustained attention but has minimal storage or retrieval components (Miller et al., 2009). Therefore, in data analysis, 0‐back can be used as a covariate to control for sustained attention statistically, allowing the analysis to focus on the storage, recall, inhibition, and updating elements of WM.

Evans and colleagues (2011) used electroencephalogram to assess speed of processing, P3 amplitude, and P3 latency in 10 adolescents with DLD and 10 age‐matched controls as they performed an *N*‐back task. There were no significant group differences in accuracy during the 1‐back task, but the children in the DLD group presented significantly lower P3 amplitude relative to matched controls in both 1‐back and 2‐back tasks. Lower P3 amplitude in the DLD group was interpreted as indicating a deficit in encoding and the need for greater cognitive resources to maintain and inhibit information.

### 1.2 Neural regions that contribute to WM and language

Several neuroimaging studies using *N*‐back tasks have identified active core areas in the prefrontal and parietal regions that are widely recognized as critical areas for executive functioning and WM in children and adults (Eriksson et al., 2015; Fishburn et al., 2014; Miró‐Padilla et al., 2020; Owen et al., 2005; Rottschy et al., 2012; Yaple & Arsalidou, 2018). Parietal areas, specifically the inferior parietal lobule (IPL), have been linked to encoding, phonological storage, and manipulation during WM tasks. Furthermore, studies have found the left dorsolateral prefrontal cortex (DLPFC) plays an important role in WM performance by filtering and monitoring information in both visual and auditory modals (Barbey et al., 2013; Cole et al., 2012: Crottaz‐Herbette et al., 2004; Lamichhane et al., 2020; Rodriguez‐Jimenez et al., 2009; Rottschy et al., 2012).

Functional imaging studies conducted on individuals with DLD have reported abnormalities in the prefrontal cortex (Gallagher & Watkin, 1997; Gauger et al., 1997) and decreased activation in left frontal and temporal language areas and subcortical areas such as the caudate and putamen while individuals performed a variety of tasks including verbal WM, task switching, covert auditory response naming, and phonological processing (see reviews: Badcock et al., 2012; de Guibert et al., 2011; Mayes et al., 2015; Pigdon et al., 2020; Weismer et al., 2005). For example, Weismer et al. (2005) used a modified listening span task to investigate verbal WM in 16 adolescents with and without DLD. Behavioral results showed children with DLD had worse accuracy and displayed longer response times for high encoding items compared to the control group. The fMRI results revealed significant hypoactivation during encoding in the left parietal region, inferior frontal gyrus, and the precentral sulcus in the DLD group. Weismer and colleagues noted the important role of the parietal region, inferior frontal gyrus, and the precentral sulcus in attentional control, language processing, and retention. Thus, hypoactivation in these regions in DLD samples provides evidence for atypical brain activation during sentence encoding and recognition. Despite the inconsistencies in the literature, there is general agreement that key language areas (frontal, temporal, and parietal cortex) are affected in individuals with DLD and overlap with brain areas active in executive function tasks such as the *N*‐back (Rottschy et al., 2012; Yaple & Arsalidou, 2018). Taken together, the left frontal and parietal areas seem important to probe in children with DLD given the role both brain regions play in executive functioning and language.

### 1.3 Functional near‐infrared spectroscopy

Functional near‐infrared spectroscopy (fNIRS) uses near‐infrared wavelengths to measure cerebral metabolic changes that serve as a proxy for neuronal activity. As neuronal activity increases in an area of the cortex, there is a concomitant increase of oxygenated hemoglobin (O_2_hb) and a decrease in deoxygenated hemoglobin that provides a blood oxygenated level‐dependent (BOLD) signal similar to fMRI (Boas et al., 2003; Fantini et al., 2018; Huppert et al., 2006; Lecrux et al., 2019). Several studies have demonstrated the utility of using fNIRS for language research in adults and children due to its relatively low cost, noninvasive nature, portability, and tolerance to motion (see reviews: Butler et al., 2020; Pinti et al., 2020; Yücel et al., 2017).

fNIRS has the advantage over other imaging techniques such as fMRI in that it is quiet and less restrictive and provides an environment that is more comfortable and conducive to capturing real‐world behavior (with associated neural responses) in children and in clinical populations that might find methods such as fMRI distressing (Pinti et al., 2020; Soltanlou et al., 2018). fNIRS also has a higher tolerance to movement than either fMRI or electroencephalography (Aslin & Mehler, 2005; Quiñones‐Camacho et al., 2019). Finally, Butler and colleagues (2020) point out that fMRI is particularly problematic for studying individuals who have difficulty in processing language, such as children with DLD, because it requires them to listen in a noisy environment.

Two fNIRS studies have investigated neural activity within a DLD population: one with children (Fu et al., 2016) and one with adults (Berglund‐Barraza et al., 2020). Fu and colleagues (2016) found higher oxygenated and deoxygenated hemoglobin in TD children in the bilateral perisylvian areas, specifically, right inferior frontal gyrus, left inferior posterior parietal junction, and left temporal and parietal junction, as they performed a sentence‐processing task. Berglund‐Barraza et al. (2020) used fNIRS and high‐definition transcranial direct current stimulation (tDCS) to investigate individual differences in WM training in two adults with DLD. Although this was a case study, their findings demonstrate that fNIRS could track individual differences in neural activity in the prefrontal cortex. Together, these studies suggest that fNIRS has the potential to capture differences in the hemodynamic response between TD children and children with DLD.

Although fNIRS research on DLD is sparse, fNIRS has been successfully used to study neural changes in children cognitive flexibility in preschoolers (Quiñones‐Camacho et al., 2019), mathematical cognition in primary school children (Skau et al., 2022), and WM and executive control in clinical populations such as Down syndrome (Xu et al., 2020), autism spectrum disorder (Zhang & Roeyers, 2019), ADHD (Gu et al., 2017), and patients with cochlear implants (Sherafati et al., 2022). In addition, fNIRS has been used to examine WM in adults (Baker et al., 2018; Berglund‐Barraza et al., 2019; Fishburn et al., 2014; Meidenbauer et al., 2021). fNIRS appears to be a viable tool for investigating neural activity in children with DLD as they perform an auditory WM memory task.

The present study was designed to explore the general cognitive mechanisms in WM that are often found to be impaired in children with DLD. A continuous auditory *N*‐back task was used to probe cognitive abilities such as sustained attention, storage, updating, and inhibition in monolingual TD children and children with DLD. fNIRS was used to examine the hemodynamic response patterns in the left DLPFC and IPL as children performed 0‐back, 1‐back, and 2‐back tasks. The 0‐back task was used in our analysis as a control for sustained attention. The research questions were as follows: (1) Are there differences in *N*‐back response accuracy and response time between children in the DLD and TD groups? (2) Are there changes in O_2_hb concentration values in left DLPFC and IPL as a function of group (TD, DLD) or task (1‐back, 2‐back)? (3) Do O_2_hb values in the left DLPFC or IPL predict accuracy and response time in children with and without DLD?

## 2 METHODS

### 2.1 Participants

Twenty‐eight school‐aged children between the ages of 9 and 14 years participated in the study. All the participants met the following criteria: (1) right‐handed; (2) monolingual English speakers; (3) no known neurological or psychiatric disorders; (4) normal‐range hearing sensitivity at 0.5, 1, 2, and 4 kHz (American National Standards Institute, 1997); (5) normal or corrected vision; and (6) no intellectual disorder as indicated by scaled scores of 6 or above on the symbolic memory subtest of the Universal Test of Nonverbal Intelligence. The DLD group consisted of 14 children (seven females) with a mean age of 11 years 5 months (*M* = 138.5 months, *SD* = 24.4 months) who scored 1.25 *SD* below the mean or greater on any two of four measures of receptive and expressive language (see below). The TD group consisted of 14 children (five females) with a mean age of 11 years 3 months (*M* = 136.8 months, *SD* = 21.0 months). All children in the TD group scored within the normal range on all four language measures.

Recruitment of children in both groups occurred simultaneously. Parents were informed of the study by word of mouth or by flyers that were distributed throughout the community. Children and parents were familiarized with research procedures before obtaining informed consent. Children came to the University for three separate sessions. Each session included a test battery as well as neuroimaging and eye‐tracking.

### 2.2 Measures

The test battery was designed to assess language, reading, and memory. Linguistic skills were measured using the comprehension and production subtests of the Test of Narrative Language (TNL‐2; Gillam & Pearson, 2017) and the Antonyms and Grammaticality Judgement subtests of the Comprehensive Assessment of Spoken Language (CASL‐2; Carrow‐Woolfolk, 2017). Phonological short‐term memory was assessed with the Non‐word Repetition subtest from Comprehensive Test of Phonological Processing—Second Edition (CTOPP‐2; Wagner et al., 2013). Complex sequential memory and short‐term visual memory were measured by the Symbolic Memory subtest from the Universal Nonverbal Intelligence Test (UNIT; Bracken & McCallum, 1998). The Auditory Working Memory subtest of the Woodcock–Johnson III Test of Cognitive Abilities (Woodcock et al., 2001) was used as a standard index of verbal WM span.

For the fNIRS task, we administered an auditory *N*‐back task that consisted of three blocks: 0‐back, 1‐back, and 2‐back. Children heard a stream of letters that contained “P,” “Q,” “R,” and “S.” In the 0‐back task, for each auditory item, children were asked to press the key that indicated the letter that was heard. In the 1‐back task, children were asked to press the key that indicated the letter presented one item before the current item. Finally, in the 2‐back task, children were asked to press the key whenever the item they heard was the same as the one that occurred two items before the current item. We created a continuous *N*‐back task because we wanted our hemoglobin concentration measure to reflect the extent of neural activity across the entirety of each block. To do that, we needed participants to respond to each stimulus, not just those stimuli that were followed by a tone.

**FIGURE 1:**
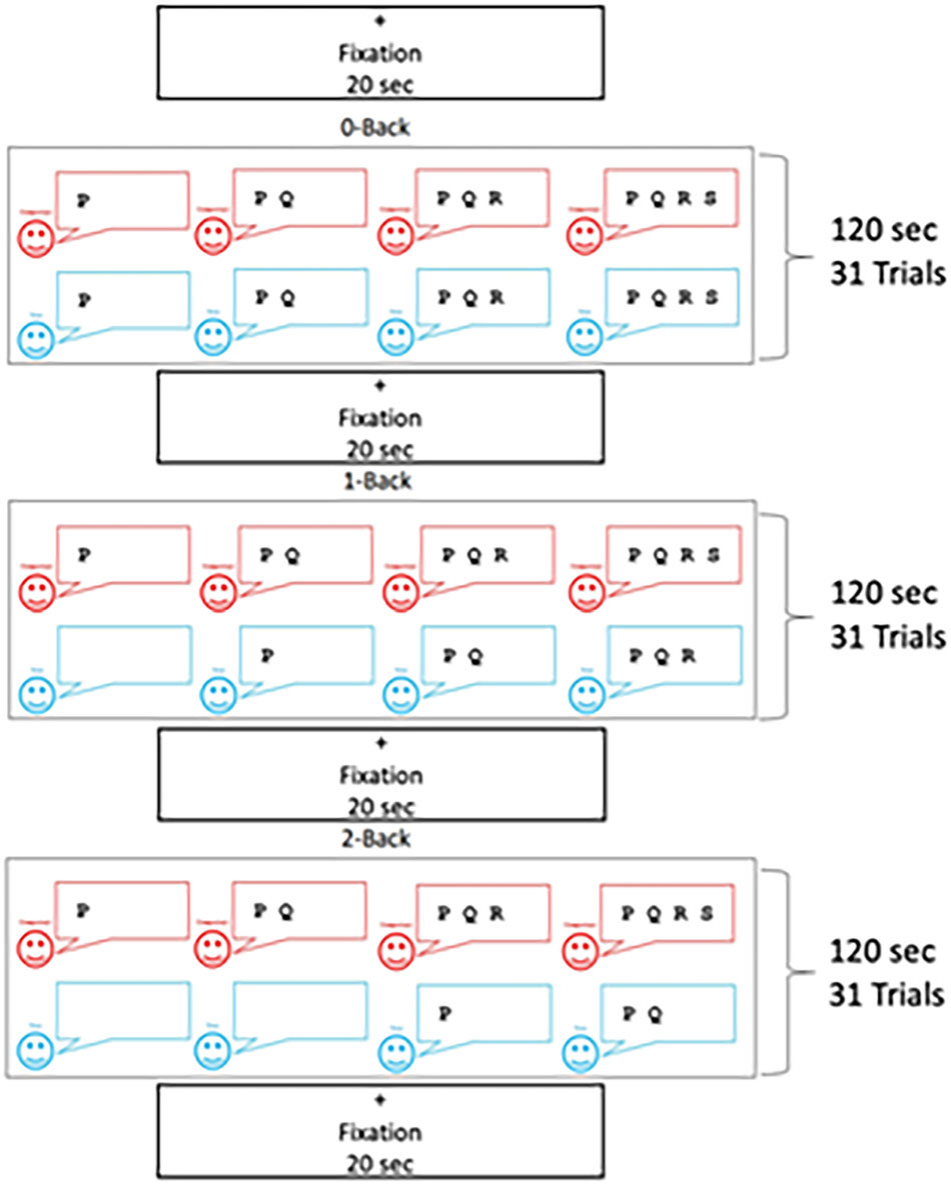
Continuous auditory *N*‐back task. During the training, children viewed a red and blue smiley face. The red smiley face represented the letter the child heard, while the blue smiley face represented the response the child should make. The smiley faces were only displayed during the training period to teach the task.

Each block contained 31 trials that were 3 s apart. Every block was proceeded with a 20‐s fixation period in which children were instructed to “look at the cross until it goes away.” The blocks were pseudorandomized and lasted a total of 120 s. The experiment was programmed in E‐prime 2.0, and children's responses were recorded on a button box that had each button marked with the letters “P,” “Q,” “R,” and “S” (see Figure 1).

### 2.3 Procedure

Participants underwent a training period in which a researcher read the instructions and provided the opportunity to practice each task before starting the actual experiment. Before the start of each block, children would receive a reminder of the instructions for the task. Once children indicated they understood the task and responded consistently to them, the researchers placed the fNIRS cap on their heads.

fNIRS data were acquired at 697.8 and 827.9 nm wavelengths using the Hitachi ETG‐4000 system (Hitachi Medical Group, Tokyo) at 10 Hz. Two 3 × 5 probe sets containing 44 channels were used for data collection. One probe set was lined up with the nasion to ensure coverage of the left and right DLPFC. The second probe was placed on the left side of the child's head with the middle detectors in the lowest row lined up with T3/T4 to ensure coverage. Regions of interest (ROIs) were determined a priori based on findings from previous *N*‐back literature as the left DLPFC (Brodmann 9/46) and IPL (Brodmann 39/40). Each ROI had between five and 10 channels.

**FIGURE 2:**
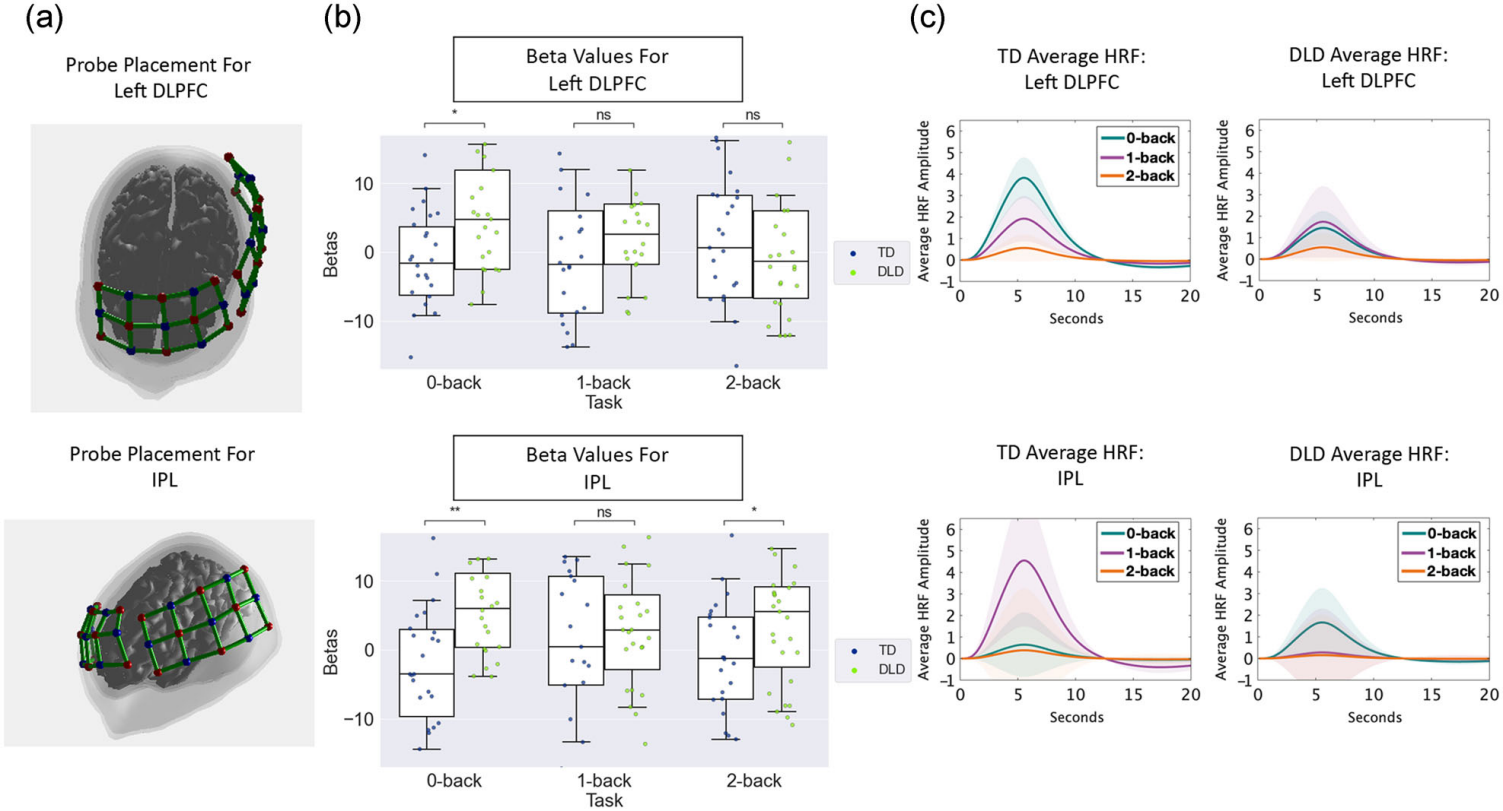
Probe placement, beta values, and average hemodynamic response function (HRF). (a) fNIRS probe placement for left DLPFC and IPL. (b) Boxplots representing the beta values for each group, task, and region. (c) HRF averaged by group and region of interest for each N‐back task. Shaded areas represent stand error. IPL, inferior parietal lobule; DLPFC, dorsolateral prefrontal cortex; DLD, developmental language disorder; TD, typically developing.

After the experimental procedure, researchers removed the optodes and obtained head measurements and channel locations via Polhemus PATRIOT 3D digitizer. Channel locations were registered to the Colin27 MRI atlas (Holmes et al., 1998) (see Figure 2a).

### 2.4 fNIRS data preprocessing

NIRS Brain AnalyzIR toolbox (Santosa et al., 2018) was used for data preprocessing and channel registration. Channels were visually inspected prior to analysis to examine overall data quality. Channels with excessive noise were removed if the coefficients of variation threshold exceeded 7.5% (Hocke et al., 2018). Next, raw intensity light values were converted to optical density values, which were then corrected for motion using the temporal derivative distribution repair procedure (Fishburn et al., 2019). The final values were then converted to oxygenated and deoxygenated concentration values using the modified Beer–Lambert law (Jacques, 2013). Oxygenated values were used as dependent values in the analyses because they have been shown to yield a more intense response to changes in physiological blood flow than deoxygenated concentration values (Strangman et al., 2002; Tachtsidis & Scholkmann, 2016). Because of the unique statistical properties of fNIRS, such as high serial correlation errors and heavy‐tailed noise distribution, an autoregressive, iteratively reweighted least‐square model (prewhitening) was used that was more statistically robust than other standard motion correction techniques (Barker et al., 2013; Huppert, 2016). Beta values were constructed by solving a generalized linear model (GLM) for every channel for each subject and task. Boxplots representing the beta values are shown in Figure 2b. The known onsets and durations of each trial throughout each scan were used to define each regressor. All regressors were then convolved with the canonical hemodynamic response function (HRF). The average HRF for group, ROI, and *N*‐back task is plotted in Figure 2c. The shaded areas represent the standard error from the function created by Martínez‐Cagigal (2022).

### 2.5 Data analysis plan

We were primarily interested in determining whether children with DLD responded slower and less accurately than their TD peers and if O_2_Hb values would differ between two conditions of *N*‐back (1‐back and 2‐back) while using 0‐back as a control. Including 0‐back measures as a fixed effect for linear mixed models allowed us to equate groups on sustained attention. After examining basic descriptive indices and correlations among measures, we computed a series of (generalized) linear mixed‐effects models that evaluated the fixed effects of the group (DLD, TD) between‐subjects factor and the task (1‐back, 2‐back) within‐subjects factor, controlling for 0‐back scores for the dependent measures of accuracy and correct response time. We used a binomial distribution with a logit link to model accuracy and a Gaussian distribution with an identity link for the correct response time. We also assessed neural activity, represented by O_2_Hb. Fixed effects for each model included between‐subject factor group (DLD, TD) and within‐subject factors ROI (IPL, left DLPFC), task (1‐back, 2‐back), and O_2_Hb beta values for 0‐back. A Gaussian distribution with an identity link was also used to model O_2_Hb beta values.

Taking a bottom‐up approach to modeling, we started with a simple model and added more complexity (main effects and interactions) with each subsequent model. Models included a random intercept for individual participants. The best‐fitting model was determined using a series of likelihood ratio tests; *p*‐values for specific effects were calculated using the Satterthwaite approximation for the degrees of freedom (Kackar & Harville, 1984; Satterthwaite, 1941). Standardized coefficients, which are similar to Cohen's *d* because they represent the standard deviation of change in the outcome for a change in the predictor (Lorah, 2018), were used as a measure of effect size. Assumptions for each model were assessed for problematic patterns, with all models showing adequate patterns. All linear mixed‐effects analyses were conducted in R 4.0.1 (R Core Team, 2020) using the lme4 (lme4 package, version 1.1‐19; Bates et al., 2015), lmerTest (Kuznetsova et al., 2017), parameters package (Lüdecke et al., 2020), and performance packages (Lüdecke et al., 2021).

## 3 RESULTS

### 3.1 Descriptive data

Descriptive measures of the language and cognition data for the two groups and Cohen's *d* effect sizes are presented in Table 1. Children in the DLD group, while being very similar in age (*p* = .83, *d* = 0.08), performed significantly more poorly than their TD controls (with large effect sizes) on each language measure: CASL Antonyms (*t*(26) = 3.53, *p* = .002), CASL Grammaticality Judgement (*t*(26) = 3.56, *p* = .002), TNL‐2 Comprehension (*t*(26) = 4.1, *p* < .001), and TNL‐2 Production (*t*(26) = 2.87, *p* = .008); and on each memory measure: Nonword Repetition (*t*(26) = 4.44, *p* < .001), Auditory Working Memory (*t*(25) = 4.32, *p* < .001), and Unit Symbolic Memory (*t*(26) = 2.91, *p* = .007). These results indicate that children in the DLD group had poor language comprehension, language production, and WM abilities compared to their TD peers.

**TABLE 1:** Summary of cognitive and language measures

	TD	DLD	*p*‐value	Cohen's *d*
Age (months)	136.8 (21.8)	138.5 (24.4)	.83	0.08
CASL Antonyms	37.1 (7.1)	26.8 (8.3)	.002	1.33
CASL Grammaticality Judgement	52.5 (9.7)	32.4 (18.2)	.002	1.34
TNL‐2 Comprehension	37.7 (4.6)	29.5 (5.9)	<.001	1.56
TNL‐2 Production	55.4 (9.3)	42.9 (13.2)	.008	1.07
Nonword Repetition	16.8(1.7)	11.3(4.3)	<.001	1.68
Auditory Working Memory	23.9 (6.6)	14.0 (5.0)	<.001	1.66
Unit Symbolic Memory	16.7 (4.3)	12.5 (3.3)	.007	1.10

*Note*: CASL Antonyms, antonyms subtest of the Comprehensive Assessment of Spoken Language (CASL‐2; Carrow‐Woolfolk, 2017); CASL Grammaticality Judgement, subtest of the Comprehensive Assessment of Spoken Language (CASL‐2; Carrow‐Woolfolk, 2017); TNL‐2 Comprehension, story comprehension subtest of the Test of Narrative Language (TNL‐2; Gillam & Pearson, 2017); TNL‐2 Production, story production subtest of the Test of Narrative Language (Gillam & Pearson, 2017); Nonword Repetition, phonological short term memory subtest for the Comprehensive Test of Phonological Processing (CTOPP‐2; Wagner et al., 2013); Auditory Working Memory, verbal working memory span subtest of the Woodcock–Johnson III Test of Cognitive Abilities (Woodcock et al., 2001); Unit Symbolic Memory, complex sequential and short‐term visual memory subtest of the Universal Nonverbal Intelligence Test (UNIT; Bracken & McCallum, 1998).

### 3.2 *N*‐back accuracy

Means and *SD*s were calculated for accuracy (assessed as proportion correct) on the three *N*‐back tasks. There was a clear difference in accuracy between groups and across our experimental tasks, with the DLD group performing significantly worse than the TD group for 0‐back (DLD *M* = 0.79, *SD* = 0.41 vs. TD *M* = 0.92, *SD* = 0.27; Cohen's *d* = 0.60), 1‐back (DLD *M* = 0.43, *SD* = 0.50 vs. TD *M* = 0.68, *SD* = 0.47; Cohen's *d* = 0.51), and 2‐back tasks (DLD *M* = 0.31, *SD* = 0.46 vs. TD *M* = 0.58, *SD* = 0.49; Cohen's *d* = 0.57).

**FIGURE 3:**
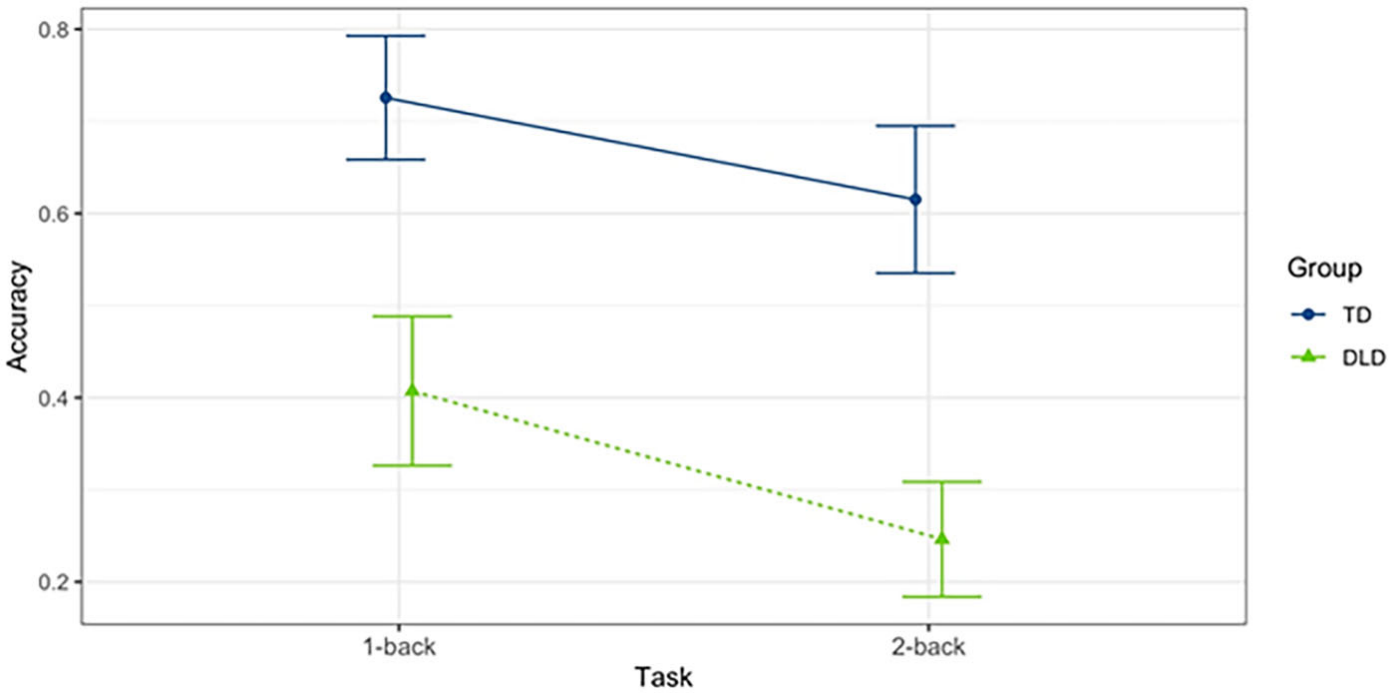
*N*‐back accuracy in the 1‐back and 2‐back conditions. Model 4 fit for two‐way interaction between group and task. DLD, developmental language disorder; TD, typically developing. Accuracy = proportion correct. Error bars represent model‐based standard errors and are, therefore, of equal width. Zero‐back is not included because it was incorporated into the model as a fixed effect.

Generalized linear mixed models were constructed for accuracy using the binomial distribution and logit link (i.e., multilevel logistic regression). This analysis was used to investigate the effects of group (DLD, TD) and task (1‐back, 2‐back) for accuracy while equating groups on sustained attention (0‐back). The first model (Model 1) was the null model that provided an estimate for the mean of accuracy without the inclusion of fixed effects. The second model (Model 2) included 0‐back accuracy and task as fixed effects. The third model (Model 3) included fixed effects for 0‐back accuracy, task, and group. The fourth model (Model 4) included a two‐way interaction between task and group with 0‐back as a fixed effect (Table 2). The best‐fitting model determined by the likelihood ratio test was Model 4 (*χ* ^2^(2) = 19.67, *p* < .001; standardized coefficient = 0.79), which contained a two‐way interaction between group and task. This model indicated that children in the DLD group had significantly lower accuracy than the TD group during 1‐back and 2‐back tasks even when we equated the groups on sustained attention (see Figure 3).

**TABLE 2:** Progression of mixed‐effects models for accuracy

	Model 1	Model 2	Model 3	Model 4
(Intercept)	−0.00 (0.27)	0.36 (0.28)	1.10^**^ (0.34)	1.04^**^ (0.34)
0‐back accuracy		−0.08 (0.07)	−0.08 (0.07)	−0.08 (0.07)
Task (reference = 1‐back)				
2‐back		−0.61^***^ (0.04)	−0.61^***^ (0.04)	−0.50^***^ (0.05)
Group (reference = TD)				
DLD			−1.46^**^ (0.47)	−1.35^**^ (0.47)
Interactions				
Task × group				−0.24^***^ (0.07)
Variance components				
Participant (Intercept)	2.01	2.10	1.56	1.57
Model summary				
Akaike Information Criteria (AIC)	20,042.64	19,730.76	19,724.54	19,715.09
Bayesian Information Criteria (BIC)	20,058.29	19,762.06	19,763.67	19,762.03
Log likelihood	−10,019.32	−9861.38	−9857.27	−9851.54
Number of observations	18,483	18,473	18,473	18,473
Number of participants	28	28	28	28

Abbreviations: DLD, developmental language disorder; TD, typically developing.

***p < .001; **p < .01; *p < .05.

### 3.3 Response time

Means and *SD*s were calculated for correct response times. The two groups were quite similar for the 0‐back (DLD *M* = 747.55, *SD* = 529.80 vs. TD *M* = 696.25, *SD* = 376.84; Cohen's *d* = 0.11) and 1‐back conditions (DLD *M* = 700.58, *SD* = 524.46 vs. TD *M* = 683.20, *SD* = 592.33; Cohen's *d* = 0.03). However, the children in the DLD group responded more slowly on the correct items in the 2‐back condition (DLD *M* = 959.98, *SD* = 592.22 vs. TD *M* = 701.76, *SD* = 652.58; Cohen's *d* = 0.41).

**FIGURE 4:**
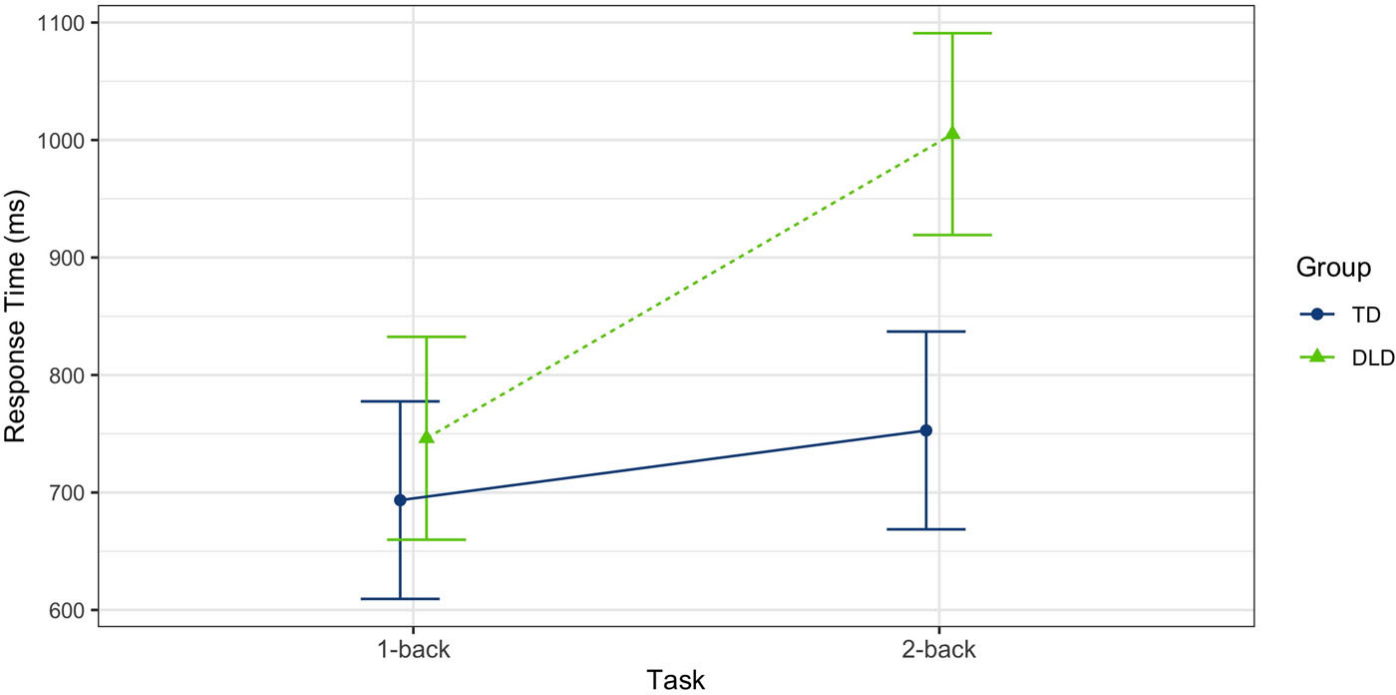
Response time for correct responses. Model 4 fit for two‐way interaction between group and task. Response time in milliseconds (ms). DLD, developmental language disorder; TD, typically developing. Error bars represent model‐based standard errors and are, therefore, of equal width.

A series of linear mixed models were computed to assess the effects of group (DLD, TD) and task (1‐back, 2‐back) for correct response time while equating groups on sustained attention (0‐back). Construction of the models followed the same blueprint as the accuracy data: Model 1 represented the null model that contained the mean estimate for correct response time without the inclusion of fixed effects. Model 2 included 0‐back correct response time and task as a fixed effect, while Model 3 included 0‐back correct response time, task, and group as fixed effects. Model 4 contained a two‐way interaction between group and task with 0‐back correct response time as a fixed effect (Table 3). The best‐fitting model, Model 4, as determined by the likelihood ratio test (*χ* ^2^(1) = 54.08, *p* < .001; standardized coefficient = 0.33), included a significant two‐way interaction between group and task. This model indicated that children in the TD group responded significantly faster than children in the DLD group during the 2‐back task, while children in both groups responded with similar speed for the 1‐back task (See Figure 4).

**TABLE 3:** Progression of mixed‐effects models for response time

	Model 1	Model 2	Model 3	Model 4
(Intercept)	824.25^***^ (61.91)	713.83^***^ (63.03)	634.74^***^ (84.45)	666.78^***^ (85.07)
0‐back RT		0.05^*^ (0.02)	0.05^*^ (0.02)	0.04^*^ (0.02)
Task (reference = 1‐back)				
2‐back		119.94^***^ (12.48)	119.72^***^ (12.48)	59.32^***^ (14.90)
Group (reference = TD)				
DLD			160.96 (118.88)	52.67 (120.50)
Interactions				
Task × group				199.58^***^ (27.09)
Variance components				
Residual	275,132.75	271,327.50	271,328.96	269,517.27
Participants (Intercept)	105,917.10	102,844.60	96,216.16	97,432.01
Model Summary				
AIC	141,029.01	125,256.83	125,257.06	125,204.97
BIC	141,050.38	125,291.86	125,299.10	125,254.01
Log likelihood	−70,511.51	−62,623.42	−62,622.53	−62,595.49
Number of observations	9171	8152	8152	8152
Number of participants	28	28	28	28

Abbreviations: DLD, developmental language disorder; RT, response time; TD, typically developing.

***p < .001; **p < .01; *p < .05.

### 3.4 fNIRS data

**FIGURE 5:**
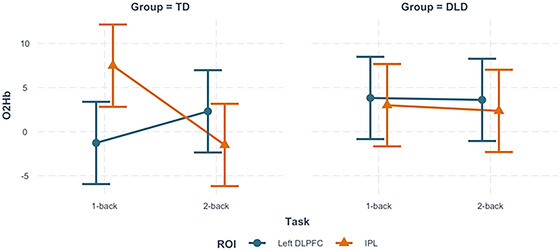
Best fitting model for O_2_hb. Model 5 fit for three‐way interaction between task, group, and ROI. On the *y*‐axis, zero represents a predicted value of zero for a specific combination of task, group, and region when they are at the mean level of 0‐back (across all random effects). O_2_hb, oxygenated hemoglobin; DLD, developmental language disorder; TD, typically developing; ROI, region of interest; IPL, inferior parietal lobule; DLPFC, dorsolateral prefrontal cortex. Error bars represent model‐based standard errors and are, therefore, of equal width.

Linear mixed models were used to assess the effects of group (DLD, TD), task (0‐back, 1‐back, 2‐back), and ROI (IPL and left DLPFC) for O_2_Hb beta values. Model 1 was the null model that provided a mean estimate of O_2_Hb beta values without the inclusion of the fixed effects. Model 2 included 0‐back O_2_Hb beta values and task as fixed effects. Model 3 included 0‐back O_2_Hb beta values, task, and group as fixed effects. Model 4 included a two‐way interaction between task and group with ROI and 0‐back O_2_Hb beta values as fixed effects. Finally, Model 5 included a three‐way interaction between task, group, and ROI, with 0‐back O_2_Hb beta values as a fixed effect (Table 4). The best‐fitting model, Model 5, determined by the likelihood ratio test, contained a three‐way interaction between task, group, and ROI (*χ* ^2^(3) = 311.54, *p* < .001; standardized coefficient = 0.83) with 0‐back O_2_Hb beta values as a fixed effect. This model indicated that O_2_Hb concentration values were influenced by task, group, and ROI together. For children in the TD group, the IPL showed an increase in O_2_Hb concentration values during the 1‐back task relative to the 2‐back. Conversely, in the left DLPFC, O_2_Hb concentration values increased during the 2‐back relative to the 1‐back task. For children in the DLD group, activation across ROIs remained similar regardless of task (see Figure 5).

**TABLE 4:** Progression of mixed‐effects models for fNIRS

	Model 1	Model 2	Model 3	Model 4	Model 5
(Intercept)	2.48 (1.67)	3.11 (1.68)	2.32 (2.36)	2.53 (2.36)	−1.47 (2.38)
0‐back beta value		0.04^**^ (0.01)	0.04^**^ (0.01)	0.04^**^ (0.01)	0.05^***^ (0.01)
Task (reference = 1‐back)					
2‐back		−1.57^***^ (0.27)	−1.57^***^ (0.27)	−2.70^***^ (0.39)	3.58^***^ (0.54)
Group (reference = TD)					
DLD			1.59 (3.33)	0.45 (3.34)	5.10 (3.37)
ROI (reference = left DLPFC)					
ROI IPL				0.72^**^ (0.27)	8.76^***^ (0.54)
Interactions					
Task × group				2.27^***^ (0.55)	−3.80^***^ (0.76)
Task × ROI IPL					−12.57^***^ (0.76)
Group × ROI IPL					−9.58^***^ (0.76)
Task × group × ROI IPL					12.14^***^ (1.07)
Variance components					
Residual	135.22	134.45	134.45	133.99	128.27
Participants (Intercept)	77.36	77.61	76.98	76.98	77.25
Model summary					
AIC	55,660.39	55,623.63	55,625.41	55,605.22	55,299.67
BIC	55,681.03	55,658.02	55,666.67	55,660.23	55,375.32
Log likelihood	−27,827.20	−27,806.82	−27,806.70	−27,794.61	−27,638.83
Number of observations	7168	7168	7168	7168	7168
Number of participants	28	28	28	28	28

Abbreviations: DLD, developmental language disorder; DLPFC, dorsolateral prefrontal cortex; fNIRS, functional near infrared spectroscopy; IPL, inferior parietal lobule; ROI, region of interest; TD, typically developing.

***p < .001; **p < .01; *p < .05.

#### 3.4.1 Secondary analyses

**FIGURE 6:**
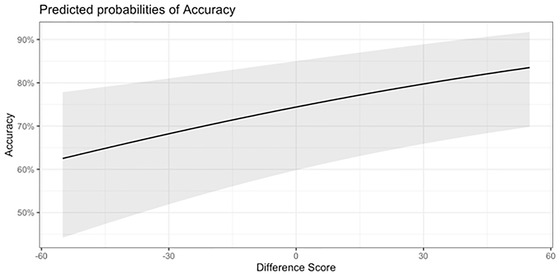
Predicted probabilities of accuracy. This model shows the trial‐by‐trial estimates of the relationship between difference scores and accuracy. The difference score was computed by subtracting inferior parietal lobule (IPL) beta values from the left dorsolateral prefrontal cortex (DLPFC) beta values.

We wondered about the extent to which performance on the *N*‐back task depended on the O_2_Hb concentration values in left DLPFC or IPL. We performed two secondary analyses—one was based on trial‐by‐trial accuracy data and the other was based on trial‐by‐trial response time data. Critical to this secondary analysis, a difference score was computed by subtracting IPL beta values from the left DLPFC beta values. We hypothesized that greater activation in the left DLPFC (relative to IPL) would be associated with more efficient filtering and inhibition of relevant information, and thus improved accuracy. This hypothesis was tested using linear mixed‐effects models, with the difference score as the main fixed effect, accounting for trial number, group, task, and 0‐back accuracy or response time, with random intercepts by individual participant. This specification allowed the investigation of trial‐by‐trial estimates of the relationship between difference scores and accuracy or response time. As with previous analyses, the accuracy models used a binomial distribution and logit link, and response time models used a Gaussian distribution and an identity link. The best‐fitting model (Model 2) for accuracy revealed a significant effect for ROI (*χ* ^2^(1) = 8.64, *p* = .003; standardized coefficient = 1.18) (Table 5). This model indicated that when children had higher O_2_Hb concentration values in the DLPFC relative to IPL, overall accuracy was increased regardless of group or task (see Figure 6). For response time, no significant effects were found as the models were not significantly different from the null model (*χ* ^2^(1) = 0.854, *p* = .355).

**TABLE 5:** Progression of mixed‐effects models for predictive accuracy

	Model 1	Model 2	Model 3
(Intercept)	1.07^**^ (0.37)	1.12^**^ (0.37)	1.12^**^ (0.37)
Task (reference = 1‐back)			
2‐back	−0.62^***^ (0.08)	−0.69^***^ (0.09)	−0.69^***^ (0.09)
Group (reference = TD)			
DLD	−1.44^**^ (0.47)	−1.48^**^ (0.48)	−1.49^**^ (0.48)
0‐back accuracy	−0.06 (0.16)	−0.06 (0.16)	−0.06 (0.16)
diffROI		0.16^**^ (0.06)	0.18^*^ (0.08)
Interaction			
Task × diffROI			−0.02 (0.10)
Variance components			
Participant (Intercept)	1.50	1.54	1.55
Model summary			
AIC	3735.53	3728.89	3730.83
BIC	3766.21	3765.70	3773.78
Log likelihood	−1862.77	−1858.44	−1858.42
Number of observations	3414	3414	3414
Number of participants	28	28	28

Abbreviations: diffROI, difference score between left dorsolateral prefrontal cortex and inferior parietal lobule; DLD, developmental language disorder; TD, typically developing.

***p < .001; **p < .01; *p < .05.

## 4 DISCUSSION

We measured neural activity during a continuous *N*‐back task in children with DLD and TD children while controlling for sustained attention (0‐back). As expected, we found that accuracy decreased and response time increased from the 0‐back to 1‐back to 2‐back conditions for children in both groups, demonstrating that the difficulty of our continuous *N*‐back task increased as a function of *N*. There were significant group differences in accuracy and response time. Children in the DLD group exhibited overall lower accuracy scores than children in the TD group and longer response times for correct responses during the 2‐back task. fNIRS data for the DLD group revealed no significant effects of task or ROI. However, left DLPFC and IPL were sensitive to task difficulty in the TD group, with decreasing O_2_Hb concentration levels in the IPL from the 1‐back task to the 2‐back tasks and increasing O_2_Hb concentration levels in the left DLPFC from the 1‐back to the 2‐back tasks. The pattern of neural results for the DLD group was significantly different, with similar levels of O_2_Hb concentration in both regions during the 1‐back and 2‐back tasks. The left DLPFC was found to be an important predictor of overall task performance.

Our results suggest a relationship between DLD and difficulties in engaging neural activity required to successfully perform the filtering, storing, and updating memory functions that underlie *N*‐back success.

There are two findings suggesting that the 2‐back task was unusually difficult for children with DLD. First, they were significantly less accurate than the children in the TD group (DLD *M* = 0.31 vs. TD *M* = 0.58). Second, they were significantly slower in responding even when they were correct (DLD *M* = 959.98 ms vs. TD *M* = 701.76 ms). It has been proposed that a minimum threshold of activation patterns in the frontal cortex is needed to support accuracy during executive function tasks (Aghajani et al., 2017; Mandrick et al., 2013, 2016; Mayes et al., 2015; Meidenbauer et al., 2021). This interpretation would be in line with Meidenbauer and colleagues (2021) and others (Aghajani et al., 2017; Mandrick et al., 2013, 2016) who reported a nonlinear effect of difficulty on activity in the frontal cortex. Specifically, frontal activity in the TD group tended to increase as a function of difficulty as long as the task did not consistently exceed the range in which the participant could still be successful at least 60% of the time. It is important to note that this interpretation is limited because we did not collect self‐report data or provide feedback to the participants, which would better corroborate this hypothesis.

For the children in the DLD group, it is possible that the lack of differences in neural activity in frontal and parietal regions during 1‐back and 2‐back tasks could relate to the heterogeneous linguistic and cognitive profile of the DLD population. Recall that children with DLD often present deficits in one or more areas of language (e.g., semantics, syntax, and morphology) together with deficits in one or more cognitive functions (e.g., WM, attention, speed of processing). Such co‐occurring deficits can impact the way children learn, produce, and comprehend language (Archibald, 2017; Badcock et al., 2012; Brown et al., 2014; Gray et al., 2019; Pigdon et al., 2020; Tomas & Vissers, 2019). As language develops, children with linguistic and cognitive deficits may employ compensatory strategies that could influence neural activity patterns in some or multiple brain regions (Badcock et al., 2012; Lee, Dick, et al., 2020; Lee, Nopoulos, et al., 2020). For example, Sherafati and colleagues (2022) found that adults with cochlear implants had reduced activation in the auditory cortex and increased activation in the left prefrontal cortex during a speech perception task compared to adults with normal hearing. Their findings suggest that adults with cochlear implants had learned to recruit cognitive processes via the left prefrontal cortex as a type of compensatory mechanism to help with deficits in the auditory cortex.

For children in the TD group, IPL O_2_Hb concentration values were greater than those in the left DLPFC during the 1‐back task. Recall that children in the TD group also had higher accuracy scores and faster response times in the 1‐back compared to 2‐back task. It is likely that 1‐back task performance in our study represents a “Goldilocks effect” (Kidd et al., 2012; Wilson et al., 2019), in which the task was at just the right level of difficulty to keep the children in the TD group engaged. Greater O_2_Hb concentration values in the IPL can be interpreted to mean greater neural effort in that region for encoding, processing, and storing relevant stimuli without putting a high demand on the left DLPFC for monitoring and filtering incoming information. However, when task difficulty increased (i.e., 2‐back), the TD group relied more heavily on the left DLPFC to monitor, filter, and inhibit incoming stimuli, as evidenced by an increase in O_2_Hb concentration values.

Our secondary analysis revealed that higher O_2_Hb concentration values in the left DLPFC predicted overall task accuracy. This finding is consistent with other research showing that the BOLD response in the left lateral prefrontal cortex is an important predictor of individual accuracy during the *N*‐back task (Cole et al., 2012; Lamichhane et al., 2020). Activity in the prefrontal cortex has been found to be positively correlated with interindividual differences in visual WM capacity, suggesting a contribution to filtering out irrelevant information (McNab & Klingberg, 2008). In addition, the prefrontal cortex has been shown to play a role in boosting parietal memory capacity (Edin et al., 2009).

As noted earlier, children with limited controlled attention may be taxing their WM system to continually filter and monitor irrelevant information affecting what is stored in WM (Awh & Vogel, 2008; Cowan et al., 2005; deBettencourt et al., 2019; Magimairaj & Montgomery, 2013). Thus, children who engaged the left DLPFC throughout the *N*‐back task likely had greater controlled attention, providing an advantage to process, store, and retrieve information stored in WM with better accuracy. Although the abovementioned studies were conducted using a visual *N*‐back task, literature has demonstrated that the left DLPFC is a key area of activation during auditory *N*‐back tasks as well (Crottaz‐Herbette et al., 2004; Huang et al., 2013; Rodriguez‐Jimenez et al., 2009).

Interestingly, children in the DLD group had similar response times to the TD group during the 1‐back task. However, group differences emerged as processing demands increased during the 2‐back. The maintenance of task accuracy appeared to come at a cost of slower response time for children in the DLD group during the 2‐back. This reflects a trade‐off between processing time and response time, as noted by Montgomery et al. (2009). Similar findings were reported in Evans et al. (2011), who also found children with DLD did not show slower response time until the auditory 2‐back task. Furthermore, Weismer et al. (2005) reported adolescents with DLD exhibited slower correct response times, but only during a higher complexity task.

Poor inhibitory control has been reported for children with DLD as a function of processing limitations (Larson et al., 2020; Marton et al., 2007; Poll & Miller, 2021). According to the inefficient inhibition hypothesis, children with DLD struggle to inhibit competing stimuli resulting in higher demands on WM to process both relevant and irrelevant information (Bjorklund & Harnishfeger, 1990; Marton et al., 2007). Uncertainty has also been shown to increase demands on WM (Coutinho et al., 2015). Our accuracy data indicated slower response times together with poorer accuracy in children in the DLD group during the more difficult 2‐back task. It is likely that their WM capacity limits were met, resulting in random, slow, inaccurate responses, thus reflecting the uncertainty and difficulty in processing and selecting their response. This idea is corroborated by the accuracy data showing children in the TD group were more accurate during the 2‐back task than the DLD group was during the 1‐back task.

### 4.1 Limitations

The sample size (*N* = 28) may be a limitation, although it is comparable to previous neuroimaging studies of DLD children (Badcock et al., 2012; Evans et al., 2011; Hugdahl et al., 2004; Weismer et al., 2005). Our analyses employed multilevel modeling techniques, which yield more reliable estimates than classical analyses with multiple comparison adjustments (Gelman et al., 2012). Cautious interpretation of these results is warranted until further studies can replicate and extend the findings. A second limitation of this study is that our *N*‐back task used a continuous stream of letters. Perhaps neural differences between groups would be more apparent in other types of complex WM tasks, such as a listening span task, that involve word or sentence recall after a secondary task. It may be valuable in future work to administer a task that can tease apart attentional differences and WM capacity within a DLD population. Finally, our study relies on a number of statistical models and tests, most of which were planned a priori. The secondary analyses of the O_2_Hb concentration values in left DLPFC or IPL were not preplanned and should be considered exploratory.

## 5 CONCLUSION

To our knowledge, this is the first study that used fNIRS to compare neural activity between children with and without DLD as they performed an auditory *N*‐back task. Our behavioral findings are generally consistent with the capacity limitation hypothesis of DLD in which higher cognitive demands resulted in lower accuracy and slower response times for children in the DLD group as compared to an age‐matched TD control group. Additionally, neural activity patterns for the DLD group were consistent with decreased cognitive effort in the face of increased task difficulty. Specifically, as opposed to the children in the TD group, the children in the DLD group presented no task‐related modulation in O_2_Hb concentration values in the IPL and the left DLPFC as a function of task difficulty. The left DLPFC was a significant predictor of accuracy across groups, supporting the role of the prefrontal cortex in filtering and monitoring incoming information as a memory task becomes more difficult. It appeared that children with DLD failed to recruit the left DLPFC for monitoring and filtering incoming information and the IPL for encoding, processing, and storing relevant stimuli when task difficulty increased with the 2‐back task. Though this result cannot conclusively assert that the failure was due to reduced WM capacity versus reduced engagement, it seems likely that both outcomes were at play. Regardless, this work demonstrates how neuroimaging can reveal what may be important idiosyncrasies in a clinical population that would otherwise be missed from behavioral data alone.

## CONFLICT OF INTEREST STATEMENT

The authors declare no conflicts of interest.

### PEER REVIEW

The peer review history for this article is available at https://publons.com/publon/10.1002/brb3.2895.

## Data Availability

The data that support the findings of this study are available in Digital Commons at Utah State University, DOI: https://doi.org/10.26078/zks9‐tk94.
